# Simulation-based education for building clinical teams

**DOI:** 10.4103/0974-2700.70750

**Published:** 2010

**Authors:** Stuart D Marshall, Brendan Flanagan

**Affiliations:** Southern Health Simulation and Skills Centre and Monash University, Melbourne, Australia

**Keywords:** Education, emergency medicine, patient safety, simulation, team

## Abstract

Failure to work as an effective team is commonly cited as a cause of adverse events and errors in emergency medicine. Until recently, individual knowledge and skills in managing emergencies were taught, without reference to the additional skills required to work as part of a team. Team training courses are now becoming commonplace, however their strategies and modes of delivery are varied. Just as different delivery methods of traditional education can result in different levels of retention and transfer to the real world, the same is true in team training of the material in different ways in traditional forms of education may lead to different levels of retention and transfer to the real world, the same is true in team training. As team training becomes more widespread, the effectiveness of different modes of delivery including the role of simulation-based education needs to be clearly understood. This review examines the basis of team working in emergency medicine, and the components of an effective emergency medical team. Lessons from other domains with more experience in team training are discussed, as well as the variations from these settings that can be observed in medical contexts. Methods and strategies for team training are listed, and experiences in other health care settings as well as emergency medicine are assessed. Finally, best practice guidelines for the development of team training programs in emergency medicine are presented.

## WHY TEAMS, WHY TEAM TRAINING?

Emergency medical care, and particularly trauma care, is usually delivered by teams.[1,2] These clinical teams must deal with situations that are highly dynamic, with large volumes of rapidly changing information, and unclear or potentially conflicting goals. All the while, the team members are under a time pressure, and the consequences of making a wrong decision or undertaking the wrong action may lead to further injury or even death.[3–5]

Although in some respects health and emergency care are unique human endeavors, these same challenges to human decision making and cognition are found commonly in other industries.[6,7] Military command and control, transport, and nuclear power generation have produced a wealth of research in the last two decades that is being used to help inform how health care can best design effective team training programs.[8–10]

Effective teamwork is essential in emergency care, due to the volume of tasks that need to be completed. The tasks themselves often require a level of knowledge and skill, mandating advanced specialist training. As a result, team members coming together to work as emergency teams often have very different skills, training and experience before joining the team. Medical, nursing, and allied health workers generally undertake training in uni-professional groups in order to learn the specialized skills and knowledge needed to fulfill their roles. The focus on knowledge and “technical skills” in undergraduate and early specialist training has until recently been to the exclusion of the additional skills required to work in a team context.[11] These skills have been termed “non-technical”[12,13], “team”[14] or “crisis resource management”[15–17] skills. A classification of the competencies (knowledge, skills, and attitudes) required by individuals to work as effective team members is given in Table 1.

**Table 1:** Individual knowledge, skills and attitude competencies to work as an effective team member from Baker *et al*.[18] after Salas *et al*.[19]

Competency	Definition
Knowledge competencies	
Shared task models/Situation assessment	A shared understanding of the situation and appropriate strategies for coping with task demands
Team-mate characteristics familiarity	Knowing the task-related competencies, preferences, tendencies, strengths and weaknesses of team-mates
Skill competencies	
Mutual performance monitoring	Tracking fellow team members’ performance to ensure that the work is running as expected and that proper procedures are followed
Flexibility/adaptability	Ability to recognize deviations from expected course of events to readjust one’s actions accordingly
Supporting/back-up behavior	Providing feedback and coaching to improve performance or when a lapse is detected; assisting team-mate in performing a task; and completing a task for the team member when an overload is detected
Team leadership	Ability to direct/coordinate team members, assess team performance, allocate tasks, motivate subordinates, plan/organize, and maintain a positive team environment
Closed-loop communication / Information Exchange	The initiation of a message by the sender, the receipt and acknowledgement of the message by the receiver, and the verification of the message by the initial sender
Attitude competencies	
Team cohesion	The total field of forces that influence members to remain in a group; an attraction to the team as a means of task accomplishment
Mutual trust	A positive attitude held by the team members regarding the aura, mood, or climate of the team’s internal environment
Collective orientation	The belief that a team approach is better than an individual one
Importance of teamwork	The positive attitude that team members exhibit toward working as a team

Strong relationships have been shown to exist between team processes and outcomes. The outcomes may be seen in terms of safe care for patients although another subjective measure is by assessment of the satisfaction of the team members.[20] Several studies have shown that sub-optimal communication and teamwork can contribute to error and poor outcomes in medical emergencies.[21–23] Communication, leadership, and group decision-making are essential to determine the priorities and goals for the team. In emergencies these priorities may change rapidly as the patient’s state evolves. The completion of tasks may also need to be undertaken in a specific order, requiring coordination between individuals. In well-rehearsed teams this coordination may be “implicit” requiring little communication, however if the situation changes, or the team rarely works together, explicit communication and leadership is needed.[24] If breakdowns in teamwork and communication account for worse outcomes and are theoretically so important in ensuring safe care, then even small improvements are likely to have a positive effect in terms of outcomes.

## WHAT ARE THE FEATURES OF AN “EFFECTIVE EMERGENCY TEAM”?

**Figure 1:**
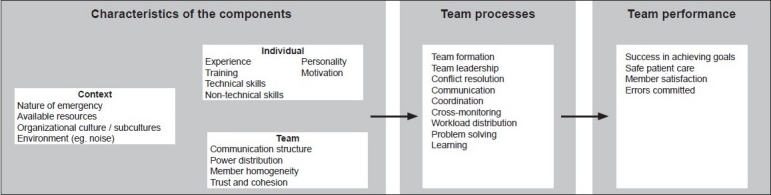
An integrated model of task performance after Salas *et al*.[20]

There are currently no generally accepted models of team performance and outcomes specific to health or emergency care. However, theoretical models are available that address team culture, behaviors, and cognition.[25–28] Salas *et al*. proposed an integrated model of teamwork in a medical setting[20] comprising input, process, and outcome measures in the context of the healthcare environment [Figure 1]. Not all of the factors in this model are amenable to change by team training alone, but it is worth considering these additional factors and how they might contribute to improvement in outcomes if they are addressed. This framework also illustrates the interactions and constraints in terms of the two targets for team training: the individual health professional, and the team in which they work. Strategies targeting the individual within the team have concentrated on cognitive approaches to train team skills, whereas team-based strategies also include fostering interpersonal relationships, a team working climate, and strengthening cohesion within the team. There is a substantial overlap in these approaches, but the focus on the “unit” of training as individual or team is an important distinction when considering methods of training.

An “effective trauma team” is “…*one where each team member knows their role and are confident in carrying out their tasks in a coordinated way towards a shared goal.*”[29] The barriers to effective trauma team function can be examined by interviewing team members; observing performance in real or simulated conditions; and reviewing adverse events to understand the task requirements and resources, and by engaging the management to help understand the broader organizational constraints.[30–32]

Interviews of trauma team members reveal that difficulties in achieving this aim in a trauma context are the result of challenges such as:[29]

1. Maintaining situation awareness
2. Leading disparate teams (including disciplines outside of emergency medicine or nursing)
3. Lack of feedback during the resuscitation
4. Lack of feedback (debriefing) after the resuscitation

Analyses of team performance in simulated and real world settings are only now starting to emerge in emergency medicine. Grissom *et al*. attempted to devise a scoring system for use in a trauma setting using ideal behavior sets identified by trauma team members.[33] The aim of the scoring system was to guide teams and team members in improvement of their processes during resuscitation. The tool would therefore allow diagnosis of team process problems, a guide for post-event debriefing and provide an indicator for where future training should be concentrated. Initial reliability measures using this tool have been variable, suggesting that further modification may be required before it is ready for widespread use.[34,35] The MedTeams project identified five “dimensions” of team performance.[36] These were based on a combination of interview data and reviews of previous incidents, and so are limited to some extent by hindsight bias. The dimensions are: (1) maintain team structure and climate, (2) apply problem solving strategies, (3) communicate with the team, (4) execute plans and manage workload, and (5) improve team skill. Each of these dimensions has underlying actions that can be measured and prioritized during training.

More general models of team function have identified five main factors from the literature that are commonly deficient in poorly performing teams.[37] These five factors are often referred to as “the big 5 of teamwork,” and consist of (1) team leadership, (2) mutual performance monitoring, (3) back-up behavior, (4) adaptability, and (5) team orientation.

1. *Team leadership*. It is a logical proposition that if tasks must be dynamically assigned and prioritized, a leader must be present and be able to have an overall view to perform this role effectively. However the leader’s role is not just one of resource allocation: effective leaders establish and maintain a positive atmosphere of collaboration, motivation, and feedback. They are also able to update team members to maintain their situation awareness and encourage questioning and suggestions.[38] Team leadership has been shown to be critical for the effective functioning of trauma teams in observational and interview studies.[34,39]
2. *Mutual performance monitoring*. Effective team members are able to monitor other team members’ performance to check if there are errors or difficulties, even whilst performing their own tasks.[26] Often however a lack of assertive behavior prevents the team members from speaking up.[40]
3. *Back-up behavior*. This is an extension of mutual performance monitoring to help prevent or mitigate error if another team member is experiencing difficulty. The team member will assist with, or complete a task for another team member or provide feedback to assist with the task.[41]
4. *Adaptability*. As previously mentioned, situations and goals may change rapidly, particularly in the emergency medicine setting. Team members must be able to adapt to these changing priorities by switching tasks or strategies.[42] An example of this is in airway management, where a failure of intubation may lead to the formulation of a new plan “on-the-fly” requiring the team to adapt and communicate the new plan.[43]
5. *Team orientation*. Orientation to the team improves motivation and cooperation between members. Initiation into the group encourages the individuals to consider the other team members’ strengths and promote information sharing and others’ input. Arguably this is close to impossible if introductions and orientation to the group occurs whilst the emergency is still developing; nevertheless the formation of a trauma team with plenty of advance notice may help this to occur routinely.

Effective communication underpins all of the ideal teamwork processes. Without effective communication teams cannot develop shared mental models, make shared decisions, or dynamically adapt to their priorities. Any team-training program must address team communication issues. This component may be a primary focus of the training such as in assertiveness training, or be integrated with the other goals of the training, such as the use of closed-loop communication within the MedTeams framework (see section “Methods of Training”).

### Translation of team training models from other settings

Much of the evidence for team training comes from military and transport settings. In these contexts combat teams or flight crews commonly work together for an extended time. An extreme example is the setting of space travel: the group may be living and working together for a period of months, and there is ample opportunity to select appropriate team members in advance.[44] Health care is clearly different to the majority of these situations. In emergency medicine team members may know each other, but this is by no means the norm. Moreover they may come together for the first time to form a team and negotiate roles even as the crisis they are to manage is evolving.[45] Care must therefore be taken in terms of extrapolating lessons learned from contexts where teams are “stable” to the transient teams of emergency medical care.

Given the absence of stable teams in all but a handful of medical settings, it would seem futile to train individuals to work as though they were part of a specific group. Team training in healthcare should therefore focus on training individuals to work in team contexts rather than training the team as a unit.[46]

## METHODS OF TEAM TRAINING

Team training may take many forms. For instance, lecture-based material can be used to impart information, or critique of “trigger videos” designed with specific educational objectives can be used to promote discussion on varying teamwork topics. Repeated practice in an artificial clinical environment has been found to be particularly useful in exploring team-working behaviors.[47] These realistic settings often include sophisticated mannequins that can represent the patient and their accompanying clinical signs and pathophysiology. This method will be familiar to many readers as “simulation-based training,” although this represents just one of many forms of simulation-based education. In addition to mannequin-based training, computer (screen)-based simulation, the use of part-task trainers (i.e. physical replicas of parts of the body), patient actors, or a hybrid of these methods can be used to replicate aspects of real world situations. Simulation also includes the use of written cases and “mental simulation” of common problems,[48] games and virtual reality devices.[49] It can therefore be seen that simulation itself is, as Gaba puts it: “…a technique—not a technology—to replace or amplify real experiences with guided experiences…that evoke or replicate substantial aspects of the real world…”[50]

Simulation allows the same scenario to be repeated multiple times to the same or different participants. It allows practice without risk to the patient, in a controlled environment, where the interaction can be recorded for future review for education or research. However, the setting may not recreate the real world exactly and many of the *patient* cues that would normally be present cannot be replicated by currently available devices-such as skin temperature, facial expression, or capillary return. The cost may also be prohibitive for many users, and access may be limited to only some members of the organization due to cost and time constraints.[50]

The modes of training have often been matched with the strategies for team training [Table 2]—overlaps exist and one mode of training is rarely employed in isolation. Note also that not all of these training strategies are used in training health care teams.

**Table 2:** Methods of team training in common use and their modes of delivery[30,69]

Team training strategy	Focus	Modes of delivery
Crisis resource management (“CRM,” also “Team coordination training”: TCT)	Underlying processes of team coordination	Lecture Video critique Immersive simulation
Cross-training	An understanding of the other aspects of the team’s work	Lecture Role modeling Immersive simulation
Team self-correction training	Strategies for monitoring their own and others’ behaviors	Role play Immersive simulation
Assertiveness training	Assertive behaviors, especially in junior staff members	Role play Video critique
Perceptual contrast training	Underlying concepts of teamwork and how they are applied	Video critique
Scenario-based training	Specific behavioral objectives embedded in common situations	Screen-based simulation Immersive simulation
Guided error training	Experience and react to common errors to transfer knowledge to real work	Immersive simulation
Stress exposure training	Knowledge of sources and effects of stress in the work environment	Lecture Video critique Immersive simulation
Metacognition training	Process of decision making and potential for error – “thinking about thinking”	Lecture Screen-based simulation Immersive simulation
Team leadership training	Specific skills required to lead a team in a given situation	Lecture Video critique Immersive simulation

### Crew/crisis resource management (CRM) training

The term “CRM training” is confusing, as CRM stands for “crew resource management” in aviation, but “crisis resource management” in healthcare. Confusion also exists because the terms “team training” and “CRM training” are often used interchangeably. CRM training is a form of team training, often also referred to as “team coordination training” (TCT).[51] The main aim of CRM training is to improve the coordination skills of an individual within a team by encouraging behaviors such as closed-loop communication, information sharing, and back-up behaviors. This is usually undertaken by delivering information about ideal behaviors, often in the form of a lecture or small group discussion, leading on to deliberate practice of the behaviors in a realistic simulated scenario. After the “immersion” in a plausible situation, the team members are then able to “debrief” their performance, often with the assistance of video data recorded during the event. The debriefing allows them to reflect on their performance and how this compares and contrasts with the ideal performance of a well-coordinated team. This debrief requires a skilled facilitator who is able to guide the discussion to achieve the optimal learning from the session.[51–53]

CRM training is perhaps almost synonymous with team training due to its wide (albeit slow) uptake in aviation in particular, and its eventual transference into health care contexts. The models of CRM training have evolved since it was first initiated in the late 1970s as “cockpit resource management” and later “crew resource management” in aviation.[16] Aviation CRM courses now rely less on the psychological theories of teamwork, and more on the practical aspects of identifying and minimizing risk - so called “Threat and Error Management” (TEM).[54] In contrast to health professionals, all flight crew have basic knowledge in “human performance limitations” at a very early stage of their careers, and CRM builds on this knowledge by emphasizing how factors such as fatigue and the time-pressure of emergencies can contribute to error. Despite 30 years of experience in CRM-style team training, there are conflicting data regarding its effectiveness. Improvements in outcome data for accidents or loss of life are difficult to attribute solely to CRM training in aviation-the same is true in healthcare. Research tools to measure the effectiveness of CRM training in aviation have included safety attitudes surveys, behavioral scoring systems, and peer evaluation in simulated and real flight conditions.[55–58] Studies have generally shown positive results with the training, although in most studies there has been a great degree of variability, and the crews had often worked together multiple times, making it difficult to compare with settings such as health care. A meta-analysis of training methods across industries confirms a positive effect of CRM training, however only 3 of the 45 studies examined were undertaken in health contexts.[59]

### Cross training

In cross training the participants are given the opportunity to take on roles of other members of the team. This theoretically allows them to better understand and therefore monitor and anticipate the actions of their teammates. Using cross training the team members can see how their tasks fit and coordinate with the rest of the team to achieve the common goal.[60] Cross training can work at three levels, relating to the responsibilities of the individual’s position, how the duties of their role relate to other team members’ duties, and finally knowledge of how the duties of others interact with their role. Training involves knowledge acquisition through lectures, and then role modeling and practice in another’s role. Immersive simulation can play a useful role in achieving this.

Cross training has been found to improve the effectiveness and efficiency of teams in air-traffic control settings by allowing a deeper understanding of roles and how back-up behaviors can assist colleagues within the context of their work.[61]

### Team self-correction training

This type of training gives the team members the tools to critique their own performance and avoid potential errors. After debriefing with an experienced facilitator, the teams are then able to undertake their own debriefing sessions without external help. This type of training is useful to create a “learning culture” within the team, and to involve all members of the team to feel they can contribute to the group’s feedback. Teams that are able to reflect on successes and failures and are “pre-occupied with failure” are more likely to develop into highly performing teams.[62]

Unfortunately, due to its nature, this form of training works most effectively with a stable team or group of team members. Teams in health also struggle to find time to debrief after the event, which can create difficulties in implementing such training.

### Assertiveness training

One of the difficulties in team management is often the ability to extract all the available information from the team members. In particular, junior members of the team may feel awkward in speaking out, even when they realize they have a vital piece of information required to manage the situation. Assertiveness training provides the tools for all team members to have their concerns and ideas heard. It allows team members to offer suggestions and initiate tasks without feeling threatened or demeaned, and without undermining the authority of the team leader.[63] Assertiveness training may be undertaken using roleplay, perhaps with patient (or team-mate) actors or in simulated scenarios. This practice has been found to be essential to establish the new assertive behaviors.[63]

### Perceptual contrast training

This uses examples, often “trigger videos”, of situations and challenges the learners to detect the positive/negative aspects of the behaviors. The participants can then contrast the ideal (and non-ideal) behaviors against their own behaviors.[48] The examples shown may become increasingly subtle. These are thought to train an improved detection of potential hazards and enhanced “situation awareness”[64]

### Scenario-based training

This technique places the learners in representations of situations to elicit responses from them. The environment may be an accurate representation of the real world with an artificial patient (mannequin or patient actor), a computer representation of the situation, or combination, such as in a virtual reality simulation. The objective is to determine the ideal behaviors, both in terms of task- and team-work in managing a situation. Scenario-based training typically re-runs the same situation more than once to allow the participants to try different strategies and evaluate which strategies are particularly useful.[65]

### Guided error training, stress exposure training and metacognition training

These methods use immersive simulation to highlight deficiencies of the environment, the processes and individual members for improvement. Guided error training allows the participants to make errors and then guides them through the consequences of these errors.[66] By experiencing the potential for errors, the team members should then be better able to avoid these in the future. Stress exposure training provides information about stress and its effects, and then places the participant in a stressful situation to modify the response to such stress. Ideally the stressful situation resembles a realistic situation from the work environment to maximize transference of the improved responses to the real world.[67] Finally metacognition or “critical thinking” training specifically educates team members about decision making and cognitive biases during life-like situations.[68] The participants are taught to recognize their biases in making decisions by deliberate practice under simulated conditions.

These final three methods are less frequently used in healthcare than in other settings, however, there is evidence from settings outside healthcare to suggest their effectiveness in improving team performance [Table 2].[59]

## LESSONS FROM TEAM TRAINING IN OTHER HEALTH DISCIPLINES

The first simulation-based course in healthcare that specifically addressed team training was described by Howard *et al*. in 1992.[52] The course used CRM principles derived from aviation and applied them to the operating theatre to address the management of anesthetic crises as the Anesthesia Crisis Resource Management (ACRM) course. One criticism of the ACRM model was the use of actors (also termed “confederates”) to play the part of the surgical team. Developing scenarios that also engaged surgeons in task and team work was later achieved as Team Orientated Medical Simulation or “TOMS.”[15] Despite the development of a limited number of TOMS scenarios, the scheduling required to train multidisciplinary teams is still a common barrier to regular full team training, and its effectiveness over anesthesia team training alone has not been proven.[70] The ACRM model remains the archetypal team training course for health, and has spawned numerous similar courses for use in different settings, including emergency medicine.[71–73] Even so, there is scant evidence to suggest that CRM training in healthcare “works.” This is primarily due to the lack of studies examining the effects of CRM training in health, with evidence confined to post-course knowledge and reaction surveys, rather than evidence of behavioral or clinical outcome effects.[74,75]

A different approach has been taken in several centers for obstetric team training. Draycott *et al*. used predominantly task-focused, scenario-based instruction in the actual workplace to train all the staff within an institution.[76] This method aimed to embed the behaviors within the organization rather than the individual, by training in multi-professional teams. The outcome measures used in this study were objective measures of hypoxic ischemic encephalopathy and low Apgar scores of the newborn, the rates of both of which were effectively halved by the training. This constitutes the only patient-based outcome evidence for team training in health care to this point.

Regular mandatory team training as is seen in domains such as aviation is still unlikely in the foreseeable future in health. A national standardized team-training course has been successfully developed in anesthesia in Australia and New Zealand.[77] Completion of this course, the Effective Management of Anaesthetic Crises or “EMAC”, is one of two course options (the other being a trauma management course) that must be taken to become a specialist in these countries.

## EVIDENCE FOR TEAM TRAINING IN EMERGENCY MEDICINE

The first documented team-training course in emergency medicine was undertaken in the late 1990s by Small *et al*.[78] A limited evaluation of this course showed a trend toward improved teamwork behaviors. Similar courses were developed that were tailored to the context of the work including remote and rural emergency departments[72,73,79] and in-situ training with ongoing support.[80] These courses rated highly in perceived usefulness and change in practice, but there are little objective data to suggest improved patient outcomes.

Emergency medicine has continued with a predominantly CRM approach to team training using mannequin-based scenarios and debriefing. The MedTeams project examined the use of a Team Coordination Training course in emergency medicine, termed the “Emergency Teams Coordination Course” (ETCC).[36,81] The ETCC is initially delivered in a classroom environment; participants are then encouraged to practice the teamwork behaviors during a half-day session in a simulated environment. Behaviorally anchored ratings scales (BARS) are then used to assess and feedback the deficiencies in teamwork behaviors to the participants using a trained facilitator. The effect of the ETCC training was assessed using a quasi-experimental study of nine teaching and community emergency departments in military and civilian settings. Staff attitudes to teamwork were improved, and error rates reduced after the training, although there were no data to support improved clinical outcomes, and there were many confounders due to the nature of the study design.[82] Further behavioral effects were examined with a small study of 20 ED physicians and nurses, half of whom were trained as “stable teams” with the ETCC method and observed after a 2-week interval in a simulated environment.[83] In this study, there was a trend toward improved behavioral markers of teamwork in the trained group that did not reach statistical significance.

One of the problems of current methods of team training is a lack of validated metrics to analyze the team deficiencies and improve feedback for training that is explicitly linked to the learning objectives.[84] An event-based approach to training (EBAT) has been suggested, that directly links scenario events to learning objectives in a clear way. In this manner, each of the team-working competencies can be addressed explicitly and systematically.[85,86]

Whether team training, be it for junior staff or as part of ongoing medical education, should be mandated as part of the credentialing process is still a question for the future. The Australasian College of Emergency Medicine recently developed a simulation-based course to address the technical and behavioral aspects of emergency medicine: The Acute and Complex Medical Emergencies (ACME) course.[87] This two and a half day course may soon become compulsory for trainees in emergency medicine - it is not as yet for senior staff, but has been made very attractive to senior staff due to a significant allocation of Continuing Medical Education (CME) points by the College for undertaking the course.

## BEST PRACTICE RECOMMENDATIONS FOR TEAM TRAINING IN EMERGENCY MEDICINE

Given the effects of factors external to the individual and team to be trained, noted in figure 1, any team training program will be limited in its success if the environmental and systemic features are not taken into account as part of developing the training program[8] Burke *et al*.[10] and Salas *et al*.[88] suggest ten best practice guidelines for team training based on pre-training, training design, and post-training periods:

### Pre-training

1. Before training can begin, an analysis of the deficiencies in the teams should be undertaken. This assessment essentially acts as a training needs analysis for the training.
2. To maximize the limited time for training, particularly when using expensive modalities such as simulation, preparatory material and exercises can be initiated, such as pre-reading video critique or even role-playing exercises.
3. An examination of the organization and its teamwork culture is essential to embed the proposed new behaviors into the overall context. This can be undertaken with safety attitude surveys and discussions with senior management.

### Training design

1. The design of the team training intervention needs to be grounded in the theory of team competencies and existing models.
2. Training should concentrate on developing shared understanding of problems. This usually means training as a team rather than as individuals and in the same work context to improve team situation awareness. This may suggest an additional benefit that may be conferred by “in-situ” simulation training.
3. A positive training climate will allow a culture of further learning after completion of the training. Again this may be measured by the use of attitudes surveys.
4. Education that emphasizes the adaptive nature of teams is more effective, as it will allow the teams to adjust to changes in goal and task requirements more readily.
5. Communication between team members is essential and should be a part of training. Particular aspects of effective communication such as closed-loop communication and assertive behavior should be designed into the intervention.
6. Training should be designed to allow explicit practice of the key team competencies listed in Table 1.

### Post-training guidance

1. Evaluation of the intervention should be systematic and based on multiple sources of data such as focus groups, observational data and post-course evaluations.

## CONCLUSION

There is mounting evidence to support the use of simulation-based team training in emergency medicine. To be effective simulation-based education needs to: address individuals’ team working skills; explore actual issues of importance in the particular workplace and organizational environment; and training teams that may perform together for an extended period. Best practice guidelines are available from other industries, but wholesale adoption of these training models should be avoided due to the different ways teams form in these different contexts. Training in teams will likely become more common in the future, and may even become a mandatory requirement for ongoing practice in some areas.
